# Molecular features of *AHDC1*: insights into an overlooked gene with broad functional potential

**DOI:** 10.1007/s00439-025-02765-7

**Published:** 2025-08-22

**Authors:** Silvana Bochicchio, Aurora Mazzetti, Lorenzo Graziani, Gian Gaetano Tartaglia, Stefano Gustincich, Remo Sanges

**Affiliations:** 1 Neuroscience Area, International School for Advanced Studies (SISSA), Trieste, Italy; 2 Centre for Human Technologies (CHT), RNA System Biology Lab, Istituto Italiano di Tecnologia (IIT), Genova, Italy; 3 Centre for Human Technologies (CHT), Central RNA Laboratory, Istituto Italiano di Tecnologia (IIT), Genova, Italy

## Abstract

Despite two decades since the completion of the human genome, many genes remain poorly understood, with their functions largely unknown. Among these, *AHDC1* stands out as a top-ranking gene in the SFARI database due to its role in the rare and likely underestimated neurodevelopmental disorder, Xia-Gibbs syndrome (XIGIS). First identified in 2014 by Prof. Richard A. Gibbs and his team at Baylor College of Medicine, *AHDC1* has historically been understudied. Until July 2023, it was classified as a Tdark gene in the Pharos database, reflecting minimal knowledge of its biological function and the lack of molecular tools for its investigation. However, interest in AHDC1 has grown significantly recently as researchers have strived to uncover the mechanisms underlying XIGIS-associated phenotypes. Recognizing these advances, the Pharos database reclassified *AHDC1* as a Tbio gene in 2023, acknowledging its rising significance and the expanding body of research surrounding it. This review consolidates the latest findings on *AHDC1*, providing an in-depth examination of its genetic structure, regulatory mechanisms, and protein functions while exploring its potential roles in nervous system development and beyond. By compiling existing literature and integrating publicly available data, this review aims to illuminate the broader biological relevance of *AHDC1* and its implications for human health and disease.

## Introduction

Xia-Gibbs syndrome (XIGIS; OMIM #615829) is a rare neurodevelopmental disorder caused by de novo heterozygous mutations in the *AT-Hook DNA-Binding Motif-Containing 1* (*AHDC1*) gene (OMIM# 615790) (Xia et al. 2014; Yang et al. 2015; Jiang et al. 2018; García-Acero and Acosta 2017; Ritter et al. 2018; Cheng et al. 2019; Díaz-Ordoñez et al. 2019; Murdock et al. 2019; Yang et al. 2019; Cardoso-Dos-Santos et al. 2020; Gumus 2020; He et al. 2020; Mubungu et al. 2021; Khayat et al. 2021b). XIGIS is a phenotypically heterogeneous disorder in which patients usually present severe developmental delays with symptoms of autism spectrum disorder (ASD) (Xia et al. 2014; Yang et al. 2015; Jiang et al. 2018; Ritter et al. 2018; Cardoso-Dos-Santos et al. 2020; Khayat et al. 2021b; García-Acero and Acosta 2017; Díaz-Ordoñez et al. 2019). The syndrome was genetically characterized in 2014 and, to date, more than 270 cases have been reported (Xia et al. 2014; Jiang et al. 2018; Khayat et al. 2021a). Human *AHDC1* is a protein-coding gene with a single 4.9-kb coding exon, which encodes a protein of 1,603 amino acids, containing two AT-hook DNA binding motifs and a PDZ binding domain consensus sequence (Xia et al. 2014; Yang et al. 2015; Shalaby et al. 2011). Almost all identified XIGIS-associated mutations are found within the *AHDC1* single coding exon and, in many cases, could lead to the translation of AHDC1 truncated forms, which may contribute to defective neural development, potentially explaining the neurological features of XIGIS (Khayat et al. 2021a; Chander et al. 2022; Gumus 2020; Ritter et al. 2018; Park et al. 2017; Wang et al. 2020). Cellular and animal models have recently been developed as biological systems for studying XIGIS (Collier et al. 2022; Li et al. 2023; Carvalho et al. 2022; Yin et al. 2023). Early findings from these models suggest the potential binding of AHDC1 to the DNA, its role in DNA methylation, and its involvement in gene expression regulation. It was hypothesized that *AHDC1* acts as a regulatory hub, facilitating interactions between enhancers and promoters to maintain gene-specific chromatin architecture (Collier et al. 2022; Kitagawa et al. 2022). Despite these emerging insights, knowledge of *AHDC1* remains very limited, while recent studies suggest that its functions are critical for mammalian development, neurodevelopmental disorders and beyond.

## *AHDC1* evolutionary appearance, genomic structure and expression regulation

**Fig. 1:**
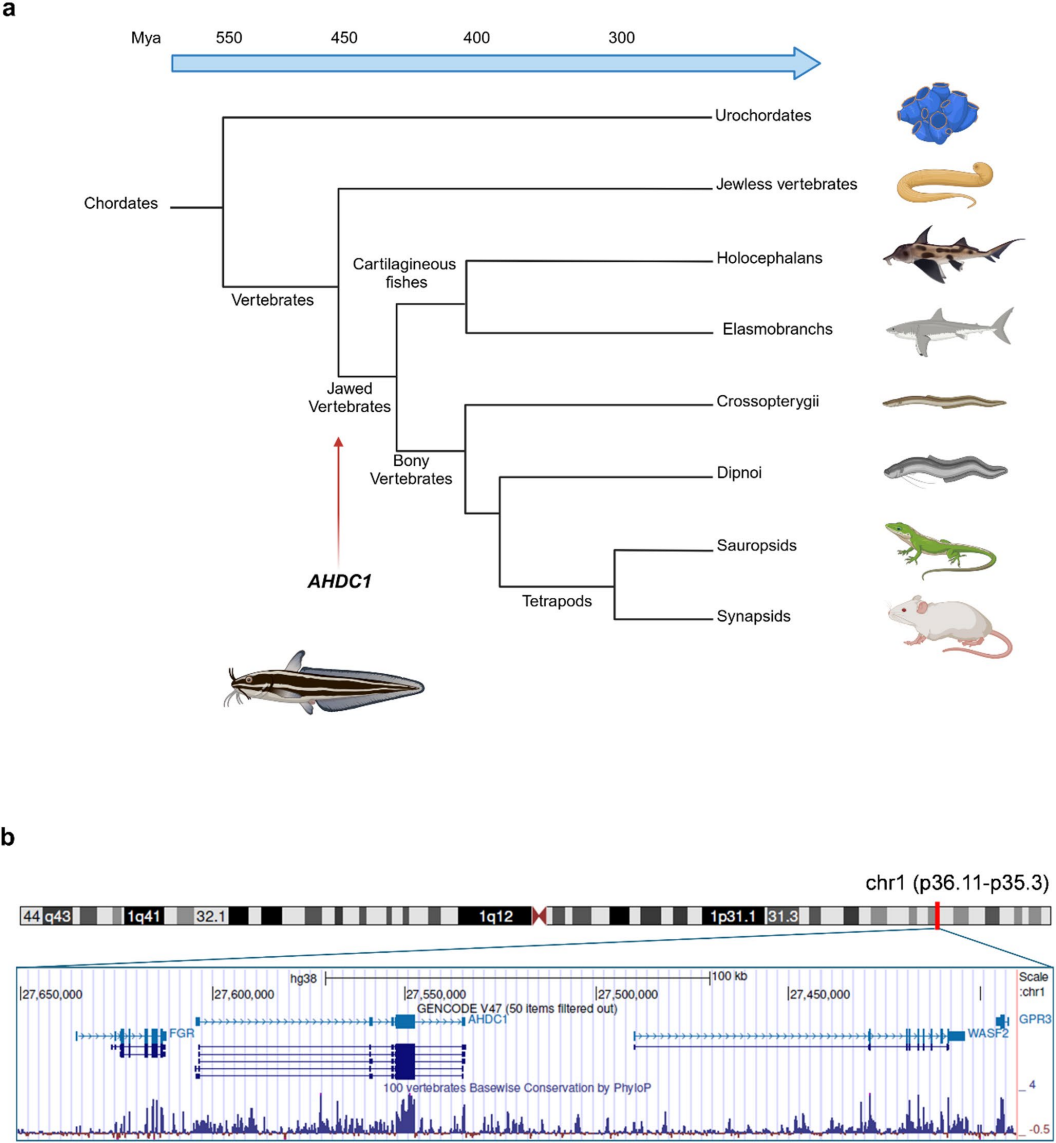
*AHDC1* evolutionary appearance and its genomic structure. **a** Simplified phylogenetic tree of the Chordates showing the first occurrence of the *AHDC1* gene in jawed vertebrates, indicated with the red arrow. Mya stands for million years ago. The diagram was created using BioRender. The Holocephalans representation is based on the elephant fish. **b** The top of the panel shows the chromosomal location of *AHDC1* with a red box highlighting the region (chr1: p36.11-p35.3) displayed in the window below. The window contains a screen capture of the UCSC Genome Browser (GRCh38/hg38), showing the structural organization of *AHDC1* transcripts, flanked by the *FGR* and *WASF2* genes, along with the gene’s conservation profile. The conservation track shows multiple sequence alignments across 100 vertebrate species, with evolutionary conservation measured using the phyloP method

To understand the evolution of *AHDC1*, we exploited the Ensembl Compara platform (Herrero et al. 2016). The *AHDC1* gene seems to have emerged in jawed vertebrates more than 450 million years ago, with its first appearance in the genome of cartilaginous fishes (Fig. 1a). The genome of the elephant shark *Callorhinchus milii* (subclass Holocephali) includes an annotated gene with a single protein-coding exon, classified as 1-to-1 ortholog to *AHDC1* in human and the other jawed vertebrates in which it has been identified. The gene exhibits 25–30% conservation at the protein level among jawed vertebrates and is located between the putative orthologs of the *FGR* and *WASF2* genes (Fig. 1b). This gene is annotated also in RefSeq (ID: XM_042338847) where it is supported by transcriptional evidence from EST and RNA-seq libraries. In UniProtKB (ID: A0A4W3K336), the corresponding protein is classified within a cluster sharing at least 50% sequence identity with two other cartilaginous fish species, *Scyliorhinus torazame* and *Chiloscyllium punctatum* (subclass Elasmobranchii), further supporting the idea that the gene originated in the common ancestor of jawed vertebrates. This occurrence coincides with a major evolutionary difference between jawed and jawless vertebrate central nervous systems (CNSs), the production of compact myelin (Yuan et al. 2018). Myelin serves as an insulating sheath around neuronal axons. This innovation enhances nerve impulse conduction speed, reduces energy consumption, and increases brain complexity and greater morphological diversity (Suminaite et al. 2019). While the specific function of *AHDC1* and its role in brain evolution remain elusive, XIGIS has already been associated with delayed myelination, raising intriguing questions about whether AHDC1 contributes to myelination and other aspects of CNS development (Jiang et al. 2018). The *AHDC1* intronless coding structure has been mostly preserved across species, with no intron additions in any of the more than 100 vertebrate species listed in Ensembl. In addition, the gene is consistently found in a region maintained syntenic across all the species, located between the *FGR* and *WASF2* genes (Fig. 1b). The preservation of the unique coding exon, together with the conserved synteny of the *locus*, suggests the existence of important regulatory features in the gene *locus*, inviting further exploration into the evolutionary history and functional roles of *AHDC1* and its genomic *locus* in vertebrate biology.

In the human genome, *AHDC1* is located on the short arm of chromosome 1 within the cytogenetic band 1p36.11-p35.3. By integrating and comparing gene-specific information from multiple data sources (e.g. National Center for Biotechnology Information (NCBI) Reference Sequence (RefSeq) and Ensembl genome browsers), it appears that the *AHDC1* gene can produce different transcript isoforms. Their structural organization includes different untranslated exons upstream of a single common 4,929 bp coding exon followed by a single downstream untranslated exon (Xia et al. 2014; Yang et al. 2015) (Fig. 1b). Although the annotated transcripts differ in the number and length of non-coding exons at 5’ UTR and the length of the last untranslated exon, they all share the single coding exon, which is relatively well conserved at the nucleotide level among vertebrates. The 3’ untranslated exon also shows high conservation levels, suggesting a potential functional significance. The variability of the non-coding exons at the 5’ UTR across different isoforms suggests the existence of alternative promoters and splicing events. Quintero-Rivera et al. described a case where the disruption of this region, due to a de novo balanced translocation with a breakpoint in *AHDC1* intron 1, was associated with a 50% reduction in its expression compared to the non-mutated *locus* (Quintero-Rivera et al. 2015). The proband exhibited developmental impairments of intellectual, speech and motor skills, as well as a clinical diagnosis of Noonan-like syndrome with cardiac left ventricular outflow tract (LVOT) defects. Later, Yang et al. described two individuals with a mutation in the 5′ UTR of *AHDC1* (c.-781 C > G) leading to its increased expression (Yang et al. 2019). Both subjects presented with obstructive sleep apnea, characterized by a higher apnea-hypopnea index (AHI) and lower oxygen saturation. Annotated features in the Encyclopedia of DNA Elements (ENCODE) show that this region overlaps with peaks of DNase I hypersensitivity in human embryonic stem cells (hESCs), suggesting that this mutation might affect important regulatory elements influencing chromatin accessibility and gene expression. The conserved 3’ UTR also points to significant post-transcriptional regulation, which could be crucial for mRNA stability and translational control as a possible miRNA target. Feng and colleagues, in addition to unveiling a connection between *AHDC1* expression and cancer pathogenesis, identified a long noncoding RNA, LINC0113328, acting as a competing endogenous RNA (ceRNA) subtracting miR-4784 from the *AHDC1* 3’ UTR and therefore increasing its expression level (Feng et al. 2019). Moreover, it has been shown that the mono-allelic expression of *AHDC1* does not impact its mRNA levels, further implicating the possible existence of transcriptional and/or post-transcriptional regulatory mechanisms governing *AHDC1* expression and homeostasis (Chander et al. 2022). These insights provide a foundation for investigating how regulatory elements within these regions contribute to gene expression and function.

## *AHDC1* expression across tissues and in the brain

**Fig. 2:**
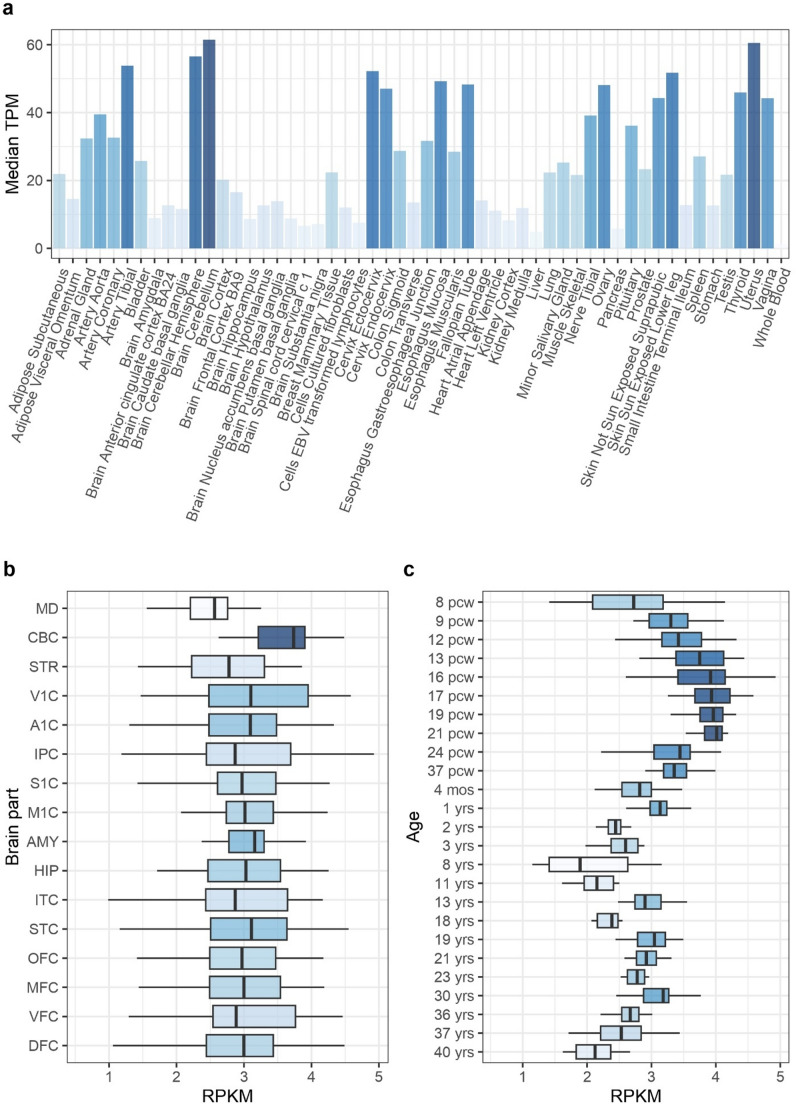
*AHDC1* expression across tissues and in the brain. **a** Expression levels of *AHDC1* from the GTEx cohort. **b** Expression levels of *AHDC1* from the BrainSpan developmental transcriptome cohort grouped by the sampled brain portion. **c** Expression levels of *AHDC1* from the BrainSpan developmental transcriptome cohort grouped by developmental time. Colour levels are determined by the expression levels, the higher the expression the darker the colour. *MD* mediodorsal nucleus of thalamus; *CBC* cerebellar cortex, *STR* striatum, *V1C* primary visual cortex, *A1C* primary auditory cortex, *IPC* posteroventral parietal cortex, *S1C* primary somatosensory cortex, *M1C* primary motor cortex, * AMY* amygdaloid complex, *HIP* hippocampus, *ITC* inferolateral temporal cortex, *STC* superior temporal cortex, *OBC* orbital frontal cortex,* MFC* anterior cortex, *VFC* ventrolateral prefrontal cortex, *DFC* dorsolateral prefrontal cortex

Analysis of public gene expression data from the Genotype-Tissue Expression (GTEx) project (GTEx Consortium 2013) reveals that *AHDC1* is widely expressed across all sampled tissues and cell lines, albeit with significant variation in expression levels, suggesting tissue-specific regulation and induction. The highest expression is observed in the cerebellum, followed by notable levels in the uterus, skin, and oesophagus (Fig. 2a) (GTEx Consortium 2013; Uhlén et al. 2015). This broad expression pattern might be associated with the phenotypic diversity seen in *AHDC1* mutations, which are linked to a neurodevelopmental disorder characterized by intellectual impairments, motor dysfunction, and effects on the skin, respiratory system, and gastrointestinal tract, with highly heterogeneous clinical presentations. Further analysis of *AHDC1* expression using data from the BrainSpan project (Jumper et al. 2021) confirms its broader expression across all brain regions and developmental stages, with the highest levels observed again in the cerebellum (Fig. 2b). Notably, when examining expression patterns by developmental stage, *AHDC1* expression shows a progressive increase from the initial sampled time at 8 post-conception weeks (pcw), peaking at 21 pcw (Fig. 2c). This timing coincides with a critical phase in human embryonic brain development, when the rhombic lip undergoes a significant expansion, producing granule and glutamatergic cells for the developing cerebellum (Haldipur et al. 2022). Experimental studies in mice corroborate the role of *AHDC1* in brain development, showing *Ahdc1* expression in the developing brain at embryonic days E11.5 and E16.5 (Quintero-Rivera et al. 2015). These stages overlap the stage of the rhombic lip expansion in humans, suggesting a conserved role for AHDC1 in cerebellar development and differentiation across species. Although more specific and comprehensive knowledge of human *AHDC1* expression remains limited, recent studies have begun to suggest potential roles in early developmental processes. AHDC1 has been implicated in early epithelial morphogenesis (Collier et al. 2022) and shows upregulation during endodermal differentiation in *GATA6*^−/−^ mutants (Heslop et al. 2022). Additionally, *AHDC1* expression is induced by retinoic acid, a pivotal signalling molecule in differentiation. Retinoic acid is critical for neuronal regulation and differentiation, including the development of cerebellar neurons, where it plays a significant role (Salero and Hatten 2007). Its activity within the neuroepithelium has been extensively studied for regulating the patterning of the hindbrain, a central nervous system region encompassing the cerebellum (Lee and Skromne 2014).

## AHDC1 functional domains, molecular functions, and cellular localization

**Fig. 3:**
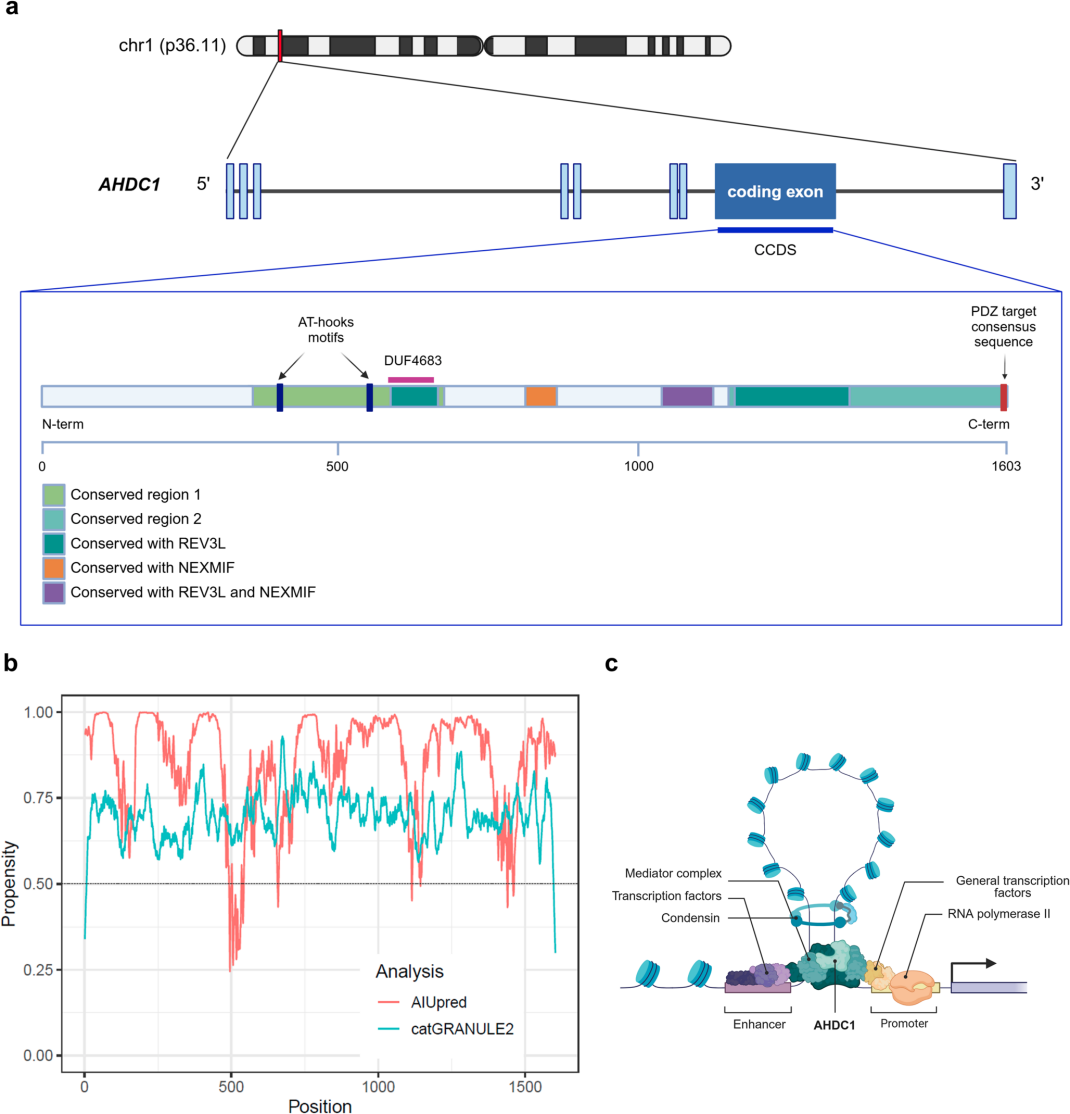
AHDC1 protein: functional domains and propensity to undergo liquid-liquid phase separation. **a** Illustration detailing the structural organization of the canonical isoform of *AHDC1*, with non-coding exons represented by light blue boxes, and the single coding exon highlighted in blue. Introns are represented as lines. The Consensus Coding Sequence (CCDS), indicated by the blue line, encodes for a protein of 1603 amino acids, schematically represented below. The protein’s features, including AT-hook domains, PDZ target consensus sequence, and conserved regions, are shown as coloured rectangles. The overlapping region with the DUF4683 domain is marked with a purple line. The illustration was generated using BioRender. **b** Predictions for AHDC1 of AIUpred (pink) on the local propensity to be disordered and of catGRANULE2 (light blue) on the local propensity to undergo phase separation. The dashed horizontal line represents the threshold defined by the tools to indicate a significant propensity for disorder or phase separation, respectively. **c** Working model of the proposed mechanism of AHDC1 gene expression control. The model was generated using BioRender

The *AHDC1* gene encodes a protein of 1,603 amino acids, which is highly conserved across vertebrates, sharing up to 94% identity between human and mouse (Khayat et al. 2021b). By aligning the human AHDC1 with the orthologs in mouse, zebrafish and western clawed frog, the conserved amino acids clustered into two regions, suggesting two main functional units; Fig. 3a summarizes all the identified putative functional units and motifs identified on the protein by Xia and collogues (Xia et al. 2014). The first conserved region contains two AT-hook DNA-binding motifs (positions 396–408 and 544–556) that are typically involved in binding to the minor groove of AT-rich DNA sequences, suggesting a role in chromatin architecture and transcriptional regulation (Xia et al. 2014; García-Acero and Acosta 2017; Karlson et al. 1989; Reeves and Beckerbauer 2001; Reeves and Nissen 1990; Huth et al. 1997). The second conserved region harbours a PDZ-binding domain at its C-terminus, which is generally associated with protein-protein interactions in cellular signalling pathways (Shalaby et al. 2011). In vitro protein-protein interaction assays have shown that AHDC1 interacts with multiple nuclear proteins, reinforcing its role as a transcriptional regulator (Collier et al. 2022; Shalaby et al. 2011; Eberl et al. 2013; Lim et al. 2006; Nozawa et al. 2010; Wong et al. 2012; Vandamme et al. 2011). Notably, AHDC1 shares conserved sequence similarities with NEXMIF, a protein associated with intellectual disability when mutated (Van Maldergem et al. 2013), and REV3L, the catalytic subunit of polymerase zeta involved in DNA repair and genome stability (Gan et al. 2008). Moreover, computational annotations report that AHDC1 contains a DUF4683 domain, a conserved but functionally uncharacterized region also present in both NEXMIF and REV3L (Paysan-Lafosse et al. 2023).

Recent studies have provided experimental evidence supporting the role of AHDC1 in epithelial development and chromatin regulation. Collier et al. demonstrated that AHDC1 acts as a key regulator of early epithelial morphogenesis by maintaining gene-specific enhancer-promoter chromatin interactions (Fig. 3c). Loss of AHDC1 leads to increased DNA methylation and reduced CTCF binding at regulatory elements, resulting in altered gene expression patterns (Collier et al. 2022). Similarly, Kitagawa et al. found that AHDC1 influences gene expression in Ewing’s sarcoma, co-localizing with BRD4 and BRG1 and potentially stabilizing their interactions with oncogenic transcription factors (Kitagawa et al. 2022). In addition to its transcriptional roles, AHDC1 may be subject to post-translational modifications. Using liquid-chromatography tandem mass spectrometry (LC-MS/MS) analysis in NCI-H1975 cells, Manetsch et al. identified AHDC1 as a target of the ADP-ribosyltransferase PARP7, a key enzyme involved in cellular stress responses and chromatin remodelling. This suggests that AHDC1 could undergo ADP-ribosylation, which might influence its function and stability (Manetsch et al. 2023). *AHDC1* has also been linked to metabolic regulation and obesity. Li et al. reported that *Ahdc1*-deficient mice exhibit increased adiposity, insulin resistance, and fatty liver, highlighting the role of the gene in energy metabolism (Li et al. 2023). Furthermore, *AHDC1* expression appears to be influenced by morphogens such as retinoic acid (RA) and bone morphogenetic protein 4 (BMP4), as well as by vitamin D signalling in the placenta, further emphasizing its regulatory importance (Ashley et al. 2022). At the cellular level, immunohistochemistry and immunofluorescence studies have demonstrated that AHDC1 is localized in the nucleus, where it is enriched in heterochromatin-rich regions near nucleoli (Uhlén et al. 2015; Thul et al. 2017; Khayat et al. 2021a). This nuclear localization pattern, combined with its interactions with chromatin-associated proteins, indicated that AHDC1 could play an important role in transcriptional regulation, genome organization, and cell differentiation.

## AHDC1 shows a strong in-silico propensity to undergo liquid-liquid phase separation

The above-mentioned studies provide a broad understanding of the potential pathways in which AHDC1 might be involved, although much remains to be discovered and/or validated. Another important issue is dictated by the protein structure, which has not been experimentally characterized yet. In the AlphaFold Protein Structure Database (Jumper et al. 2021), the majority of AHDC1 structure predictions have poor confidence scores, suggesting that most of the protein might be unstructured. Accordingly, over 70% of the protein is predicted to be disordered using tools like Espritz, MobiDB-lite and AIUPred (Walsh et al. 2012; Necci et al. 2017; Erdős and Dosztányi 2024) (Fig. 3b). Disordered protein regions are known to promote liquid-liquid phase separation (LLPS). In several cases, intrinsically disordered regions have been demonstrated to be sufficient for mediating phase separation, as observed in proteins such as hnRNPA1 (Molliex et al. 2015), hnRNPA2B1 (Xiang et al. 2015), Ddx4 (Nott et al. 2015) and Laf-1 (Elbaum-Garfinkle et al. 2015). Recently, the intrinsically disordered measles virus N protein, have also been shown to undergo LLPS and contribute to membrane-less condensates with essential cellular roles (Guseva et al. 2023). These works underscore the potential role of intrinsic disorder in facilitating LLPS and suggest that disordered regions may play a role in the formation and function of membrane-less organelles. To assess the phase separation potential of *AHDC1*, we employed the catGRANULE2 algorithm, which integrates physicochemical properties and AlphaFold2-derived features. Trained on a dataset consisting of 3333 known human LLPS proteins and 3252 non-LLPS proteins, this tool has demonstrated superior predictive performance compared to other available predictors (Monti et al. 2025). This analysis predicts that AHDC1 has a strong propensity for LLPS across its entire sequence (Fig. 3b).

Intrinsically disordered regions (IDRs) have gained increasing significance in cell biology due to their ability to mediate diverse intra- and intermolecular interactions, thereby regulating key cellular functions (Babu 2016). LLPS, induced by IDRs, has been implicated in organizing and modulating various vital biological processes, ranging from chromosome condensation, transcription, splicing, and translation, to synaptic activity, receptor activation and signalling pathways (Wang et al. 2021; Chakraborty et al. 2024). Within living cells, phase separation forms membrane-less organelles or condensates (Jin et al. 2021; Wen and Ma 2022). Notably, AHDC1 has been reported as one of the components of the paraspeckles, nuclear condensates involved in controlling gene expression (Naganuma et al. 2012). Despite this, its non-essential role in paraspeckles formation, together with our novel in-silico predictions, again prompts intriguing questions about its functions. The observations here reported support the hypothesis that AHDC1, with its predicted disordered regions and phase separation propensity, could undergo LLPS, contributing to condensate formation and possibly influencing cellular processes. Nevertheless, rigorous experimental validation will be crucial to confirm the predicted propensity of AHDC1 to undergo LLPS.

## *AHDC1* mutations

Human *AHDC1* is mutated in XIGIS subjects, and all known pathogenic mutations span most of the length of the single coding exon of the gene (Table 1). Most of these mutations are presumed truncating variants (substitutions/small indels), all predicted to be nonsense or frameshift mutations. The presence of a single coding exon suggests that mutant mRNAs may escape the nonsense-mediated decay (NMD) surveillance mechanism, resulting in the translation of possible truncated forms of the protein (Lindeboom et al. 2016; Neu-Yilik et al. 2011). This *NMD escaping hypothesis* was experimentally confirmed for some *AHDC1* mutations, by analyzing the expression of individual alleles in patient blood samples harbouring truncating mutations within the *AHDC1* coding exon (Chander et al. 2022), suggesting a dominant‐negative or a gain-of-function mechanism underlying XIGIS (Khayat et al. 2021b). Along this line, at least ten individuals were diagnosed with XIGIS based upon de novo missense mutations in *AHDC1* (Gumus 2020; Khayat et al. 2021a). The mapping of these mutations to the protein sequence identifies an overlap mostly with two regions, the second AT-hook domain and one of the portions sharing conservation with REV3L (Khayat et al. 2021b). These variants are predicted to result in full‐length protein products, providing additional support for the hypothesis of a dominant‐negative or gain-of-function pathogenetic mechanism (Khayat et al. 2021b). Individuals with large de novo deletions encompassing the entire *AHDC1* locus and diagnosed with XIGIS-overlapping features (Ritter et al. 2018; Park et al. 2017; Wang et al. 2020) have also been reported. This raises an important point because it suggests that *AHDC1* haploinsufficiency may also be a potential pathogenic mechanism at the basis of XIGIS. However, these deletions often span from tens to thousands of kilobases and include other gene *loci*, which may contribute to the XIGIS‐like phenotypes, reducing the supporting evidence for an XIGIS model based upon haploinsufficiency of *AHDC1*. Of note, a recent study by Chander et al. analyzed the RNA expression patterns in an individual diagnosed with XIGIS presenting the smallest known contiguous deletion of *AHDC1* (∼350 kb), encompassing eight other genes within chr1p36.11 (Chander et al. 2022). The authors found that *AHDC1* exhibited a mono‐allelic expression pattern with no deficiency in its overall expression levels, in contrast to the other deleted genes which exhibited a 50% reduction in mRNA expression (Chander et al. 2022). This emphasizes the possible implication of different genes in the observed phenotype. As previously anticipated, these results also suggest that *AHDC1* expression in this individual is compensated by a specific, albeit unknown, feedback regulatory mechanism (Chander et al. 2022). In addition to mutations in the coding exons of *AHDC1* associated with Xia-Gibbs syndrome, Yang et al. recently described two additional ones in the 5′ UTR of *AHDC1* (c.-88 C > T and c.-781 C > G) in patients with obstructive sleep apnea, one of the clinical manifestations of XIGIS (Yang et al. 2019) (Table 1). Notably, as previously mentioned, the mutation c.-781 C > G was also suggested to affect the expression of *AHDC1* (Yang et al. 2019).

**Table 1:** *AHDC1* mutations

DNA level	Protein level	Mutation type	Validation status	Reference
1p36.11-p35.3	NA	Microdeletion	Clinically validated	Park et al. (2017)
c.-88 C > T	NA	5’ UTR	Experimentally validated	Yang et al. (2019)
c.-781 C > G	NA	5’ UTR	Experimentally validated	Yang et al. (2019)
c.139 C > T	p.(Pro47Ser)	Missense	Clinically validated	Khayat et al. (2021b)
c.451 C > T	p.(Arg151*)	Nonsense	Clinically validated	Cardoso-Dos-Santos et al. (2020)
c.514dup	p.(Ser172Lysfs*8)	Frameshift	Clinically validated	Della Vecchia et al. (2021)
c.643dup	p.(Ser215Lysfs*16)	Frameshift	Experimentally validated	Khayat et al. (2021b)
c.730del	p.(Ile244Serfs*16)	Frameshift	Clinically validated	Fan et al. (2022)
c.750_753del	p.(Thr252Alafs*7)	Frameshift	Clinically validated	Jiang et al. (2024)
c.787del	p.(Ala263Profs*26)	Frameshift	Clinically validated	Jiang et al. (2024)
c.784 C > T	p.(Gln262*)	nonsense	Clinically validated	Jiang et al. (2018)
c.917del	p.(Pro306Leufs*146)	Frameshift	Clinically validated	Jiang et al. (2024)
c.976_988del	p.(Ser326Thrfs*122)	Frameshift	Clinically validated	Jiang et al. (2024)
c.979 C > T	p.(Gln327*)	Nonsense	Clinically validated	Murdock et al. (2019)
c.994 C > T	p.(Gln332*)	Nonsense	Clinically validated	Carvalho et al. (2022)
c.1073dup	p.(Leu359Thrfs*19)	Frameshift	Clinically validated	Jiang et al. 2024)
c.1102_1114del	p.(Cys368Alafs*80)	Frameshift	Clinically validated	Romano et al. (2022)
c.1122dup	p.(Gly375Argfs*3)	Frameshift	Clinically validated	Yang et al. (2015), Mubungu et al. (2021)
c.1155dup	p.(Arg386Alafs*3)	Frameshift	Clinically validated	Lin et al. (2022)
c.1206del	p.(Arg403Alafs*49)	Frameshift	Clinically validated	Danda et al. (2022)
c.1313del	p.(Pro438Leufs*14)	Frameshift	Clinically validated	Jiang et al. (2024)
c.1402dup	p.(Cys468Leufs*49)	Frameshift	Clinically validated	Bosch et al. (2016)
c.1446del	p.(Val483Tyrfs*16)	Frameshift	Clinically validated	Romano et al. (2022)
c.1459 C > T	p.(Arg487Trp)	Missense	Clinically validated	Khayat et al. (2021a), Gumus (2020)
c.1480 A > T	p.(Lys494*)	Nonsense	Clinically validated	Yang et al. (2015)
c.1481_1482del	p.(Lys494Serfs*22)	Frameshift	Clinically validated	Carvalho et al. (2022)
c.1529del	p.(Gly510Alafs*12)	Frameshift	Clinically validated	Díaz-Ordoñez et al. (2019)
c.1610G > A	p.(Gly537Asp)	Missense	Clinically validated	Khayat et al. (2021b)
c.1617del	p.(Met539Ilefs*46)	Frameshift	Clinically validated	(Jiang et al. 2024)
c.1642G > A	p.(Gly548Ser)	Missense	Clinically validated	Khayat et al. (2021b)
c.1646G > A	p.(Arg549His)	Missense	Clinically validated	Khayat et al. (2021b)
c.1653del	p.(Lys552Argfs*33)	Frameshift	Clinically validated	Carvalho et al. (2022)
c.1758del	p.(Lys586Asnfs*37)	Frameshift	Clinically validated	Danda et al. (2022)
c.1759 C > T	p.(Arg587*)	Nonsense	Clinically validated	Ritter et al. (2018)
c.1819G > A	p.(Asp607Asn)	Missense	Experimentally validated	Khayat et al. (2021b)
c.1881del	p.(Gln627Hisfs*105)	Frameshift	Clinically validated	Yang et al. (2015)
c.1945del	p.(Ala649Profs*83)	Frameshift	Clinically validated	Jiang et al. (2018)
c.1975del	p.(Leu659Cysfs*73)	Frameshift	Clinically validated	Salvati et al. (2022)
c.2030del	p.(Gly677Alafs*55)	Frameshift	Clinically validated	García-Acero and Acosta (2017)
c.2062 C > T	p.(Arg688*)	Nonsense	Clinically validated	Jiang et al. (2018)
c.2136del	p.(Gly714Alafs*18)	Frameshift	Clinically validated	Jiang et al. (2024)
c.2188dup	p.(Glu730Glyfs*38)	Frameshift	Clinically validated	Romano et al. (2022)
c.2188G > T	p.(Glu730*)	Nonsense	Experimentally validated	Khayat et al. (2021b)
c.2192dup	p.(Asp732Argfs*36)	Frameshift	Clinically validated	Romano et al. (2022)
c.2229del	p.(Ser744Profs*188)	Frameshift	Clinically validated	Jiang et al. (2018)
c.2260 C > T	p.(Gln754*)	Nonsense	Clinically validated	Jiang et al. (2024)
c.2373_2374del	p.(Cys791Trpfs*57)	Frameshift	Clinically validated	Jiang et al. (2018)
c.2374G > C	p.(Gly792Arg)	Missense	Clinically validated	Khayat et al. (2021b)
c.2415del	p.(Leu806Trpfs*126)	Frameshift	Clinically validated	Jiang et al. (2018)
c.2424_2425dup	p.(Gly809Valfs*124)	Frameshift	Clinically validated	Romano et al. (2022)
c.2468 C > G (50% mosaic)	p.(Ser823*)	Nonsense	Clinically validated	Romano et al. (2022)
c.2473 C > T	p.(Gln825*)	Nonsense	Clinically validated	Ritter et al. (2018)
c.2520del	p.(Arg841Alafs*91)	Frameshift	Clinically validated	Khayat et al. (2021b)
c.2529_2545del	p.(Asp845Argfs*40)	Frameshift	Clinically validated	Yang et al. (2015)
c.2547del	p.(Ser850Profs*82)	Frameshift	Clinically validated	Xia et al. (2014)
c.2565del	p.(Phe855Leufs*77)	Frameshift	Clinically validated	Jiang et al. (2024)
c.2641_2644dup	p.(Gln882Argfs*10)	Frameshift	Clinically validated	Jiang et al. (2024)
c.2644 C > T	p.(Gln882*)	Nonsense	Clinically validated	Jiang et al. (2018)
c.2691del	p.(Val898Trpfs*34)	Frameshift	Clinically validated	Jiang et al. (2018)
c.2772del	p.(Arg925Glufs*7)	Frameshift	Clinically validated	Jiang et al. (2024)
c.2773 C > T	p.(Arg925*)	Nonsense	Clinically validated	Jiang et al. (2018)
c.2849del	p.(Pro950Argfs*192)	Frameshift	Clinically validated	Romano et al. (2022)
c.2885dup	p.(Pro963Alafs*23)	Frameshift	Clinically validated	Ritter et al. (2018)
c.2889_2892del	p.(Ala964Thrfs*177)	Frameshift	Clinically validated	Cheng et al. (2019)
c.2898delC	p.(Tyr967Thrfs*175)	Frameshift	Clinically validated	Jiang et al. (2018)
c.2908 C > T	p.(Gln970*)	Nonsense	Clinically validated	Jiang et al. (2018)
c.2932 C > T	p.(Gln978*)	Nonsense	Experimentally validated	Khayat et al. (2021b)
c.3185_3186del	p.(Thr1062Serfs*63)	Frameshift	Clinically validated	Jiang et al. (2024)
c.3204 C > G	p.(Tyr1068*)	Nonsense	Experimentally validated	Khayat et al. (2021b)
c.3466 C > T	p.(Gln1156*)	Nonsense	Experimentally validated	Khayat et al. (2021b)
c.3543dup	p.(Phe1182Valfs*11)	Frameshift	Clinically validated	Jiang et al. (2024)
c.3773 C > G	p.(Ser1258*)	Nonsense	Clinically validated	Jiang et al. (2018)
c.3809del	p.(Gln1270Argfs*75)	Frameshift	Clinically validated	Yang et al. (2015)
c.3814 C > T	p.(Arg1272*)	Nonsense	Clinically validated	Popp et al. (2017)
c.3989 C > A	p.(Ser1330*)	Nonsense	Clinically validated	Jiang et al. (2018)
c.4042T > C	p.(Ser1348Pro)	Missense	Clinically validated	Khayat et al. (2021b)
c.4289dup	p.(Ala1432Glyfs*49)	Frameshift	Clinically validated	Romano et al. (2022)
c.4321 C > T	p.(Gln1441*)	Nonsense	Clinically validated	Jiang et al. (2024)
c.4370 A > G	p.(Asp1457Gly)	Missense	Clinically validated	Gumus (2020)
c.4432 C > T	p.(Pro1478Ser)	Missense	Clinically validated	Khayat et al. (2021b)
c.4438del	p.(Glu1480Lysfs*67)	Frameshift	Experimentally validated	Khayat et al. (2021b)
c.4494dup	p.(Cys1499Valfs*9)	Frameshift	Clinically validated	Ritter et al. (2018)

The table reports the mutation at the DNA level, at the protein level (if any), the mutation type, the validation status and the reference. Displayed information is taken from the indicated references

## Correlation genotype-phenotype in Xia-Gibbs patients

XIGIS exhibits a vast clinical spectrum, characterized by significant variability in age of onset, type, and severity of manifestations. This diversity, combined with the limited number of patients sharing the same variant, has made it challenging to establish specific genotype-phenotype correlations (Mubungu et al. 2021; Della Vecchia et al. 2021). While no significant associations have been identified between sex, age, or ethnicity and the manifestation of the syndrome, recent findings have started to provide insights into putative phenotypic patterns (Jiang et al. 2024). A very recent work performing latent class analysis (LCA) of 97 published mutations identified three distinct phenotypic subtypes: Ataxia, Sleep Apnea & Short Stature, and Neuropsychological. The Neuropsychological, the most frequent subtype, is characterized by high probabilities of seizures, ataxia, and autism. The Ataxia subtype primarily presents with motor coordination challenges, while the Sleep Apnea & Short Stature subtype is distinguished by obstructive sleep apnea and growth impairments. Despite this stratification, no clear correlation was observed between the *AHDC1* variant position and the identified subtypes. However, age and variant type emerged as potential predictors of subtype classification. The described work represents the most updated meta-analysis of clinically diagnosed cases associated with *AHDC1* mutations, published up to mid 2024. These cases are collected and summarized in the Table 1 of the article by N. Jiang et al. and have enabled an initial assessment of possible genotype-phenotype correlations in XIGIS (Jiang et al. 2024).

Previous studies faced multiple challenges in identifying specific genotype-phenotype correlations, often failing to reach a strong consensus. According to a work by Y. Jiang et al. in 2018, some *AHDC1* mutation positions do correlate with specific clinical observations. Variants near the C-terminal region of the encoded protein are generally associated with more severe forms of the disorder, whereas mutations closer to the N-terminal region often correlate with milder phenotypes with better cognitive outcomes (Jiang et al. 2018). Exceptions to these trends highlighted the complexity of the condition. For instance, Ritter et al. described an individual with a C-terminal mutation who exhibited only minor intellectual disability, as evidenced by the ability to speak three languages (Ritter et al. 2018). Conversely, Murdock et al. documented a case involving a nonsense mutation near the N-terminus that resulted in severe intellectual disability (Murdock et al. 2019). Similarly, Cardoso-dos-Santos et al. reported a stop-gain mutation near the N-terminus associated with a severe phenotype (Cardoso-Dos-Santos et al. 2020). Further correlations have been noted between mutation positions and specific symptoms. Seizures and scoliosis tend to be more frequently associated with truncating mutations in the N-terminal and mid-protein regions than with those at the C-terminus (Khayat et al. 2021b). Additionally, individuals with missense *AHDC1* mutations show a higher incidence of seizures compared to those with truncation mutations (Khayat et al. 2021b). These findings underscore the complex and heterogeneous relationship between genotype and phenotype in XIGIS, emphasizing the need for further studies with larger cohorts to refine classification, explore potential associations, and validate their significance and reproducibility.

## ***AHDC1*** and the association between autism and schizophrenia

ASD and schizophrenia (SZ) are two different disorders considered to have a certain degree of overlap. ASD is a neurodevelopmental disorder typically identified in early childhood, characterized by impaired social communication, restricted interests and repetitive behaviour (Liu et al. 2017). SZ is a neuropsychiatric disorder that affects approximately 1% of the world population and is characterized by psychotic symptoms such as hallucination, delusions, disjointed speech, depressive symptoms and cognitive deficits (Marder and Cannon 2019). Despite their differences, these diseases share several common characteristics such as the heterogeneous array of symptoms with a wide range of severity, the possible involvement of intellectual disability (Morgan et al. 2007), and the association with deficits in the theory of mind and impairments in mirror neuron function. Additionally, they are multifactorial and polygenic, with both genetic and environmental components contributing to their development (Owen et al. 2010; Niklasson et al. 2009; Brown 2011; Karimi et al. 2017; King and Lord 2011). *AHDC1* is classified at level 1S in the Simons Foundation Autism Research Initiative (SFARI) database, highlighting the strongest association with autism, of which several features are often reported in XIGIS subjects. Moreover, *AHDC1* has a 1S score in SFARI, indicating not only a high-confidence association with ASD but also its classification as syndromic, further supporting implication in the disease. SZ represents a risk factor for people with autism, with studies indicating that this disorder is up to six times more common in ASD patients. In addition, several copy number variations and specific genes have been associated with both conditions (Jutla et al. 2022). Finally, the literature suggests that RA signalling is involved both in schizophrenia (Reay et al. 2020) as well as autism (Zhou and Li 2018). We have identified two studies in which mutations in *AHDC1* have been in some way associated with schizophrenia, providing support to the idea that AHDC1 might also be one of the molecular factors at the basis of the aetiology of both disorders. One study reports a patient diagnosed with schizophrenia at around 13 years of age who carried a de novo nonsense mutation in the *AHDC1* gene (Cardoso-Dos-Santos et al. 2020). Another study identified 49 de novo missense mutations that are likely causative of sporadic cases of schizophrenia (Guipponi et al. 2014). In one of these patients, schizophrenia was associated with a de novo missense mutation in *AHDC1*. This case is particularly interesting because the authors report that they have excluded families with a history of autism, intellectual disability and other confounding disorders, suggesting that the patient might have presented schizophrenia without features of XIGIS.

## In vivo and in vitro models of *AHDC1* mutations

In the studies mentioned so far, murine models were established to investigate the biological implications of *AHDC1* mutation or loss. Collier et al. generated chimeric CRISPR mouse mutants and observed that mutant Gibbin caused a spectrum of developmental patterning defects. The authors claimed that both heterozygous and homozygous mutants failed to survive past birth and, therefore, the spectrum of phenotypes described in their work was related to E18 mosaic mutant embryos. They observed that the most severely affected embryos were undersized, hypovascularized, missing eyes, had open ventral walls, and showed skin stratification defects, with the epidermis appearing incompletely attached to the underlying dermis. Instead, less severe mutants displayed eyes open at birth or had craniofacial abnormalities like short snouts or craniosynostosis (Collier et al. 2022). In contrast, Li et al. were capable of generating surviving heterozygous *Ahdc1*-deficient mice (*Ahdc1*^+/−^) using CRISPR/Cas9 gene-editing technology. No homozygous null mice (*Ahdc1*^−/−^) were obtained, indicating a loss of viability in the complete loss of *Ahdc1* which, in particular, led to embryonic-lethal phenotypes after E3.5. Compared with the littermate wild-type mice (*Ahdc1*^+/+^), *Ahdc1*^+/−^ mice had significantly lower levels of *AHDC1* mRNA expression in the liver, hypothalamus, and cerebral cortex, with the most obvious phenotype related to obesity (Li et al. 2023). The homozygous disruption of *Ahdc1* leads to embryonic-lethal phenotypes, suggesting that Ahdc1 is necessary for correct development, particularly during the early stages (Collier et al. 2022; Li et al. 2023). Considering the heterozygous mice, the complete deletion of one of the two copies of *Ahdc1* leads to viable mice presenting most of the phenotypes related to metabolic disorders, indicating that a single functional copy is sufficient for survival (Li et al. 2023). Intriguingly, the chimeric CRISPR heterozygous mutant mice generated by Collier et al. failed to survive past birth (Collier et al. 2022). The differences in survival outcomes between the two studies could be attributed to the employed CRISPR/Cas9 strategies, leading to potentially distinct genetic and molecular outcomes. While in Li et al. the complete coding exon of *AHDC1* was deleted (Li et al. 2023), in Collier et al. the use of sgRNAs targeting a small internal portions of the *AHDC1* coding sequence resulted in a situation where the target exon was not completely lost and mutant protein forms could have been generated (Collier et al. 2022). Once again, this would support a gain-of-function or dominant negative mechanisms potentially contributing to the observed spectrum of developmental patterning defects in AHDC1 mutants.

In addition to the murine models, a zebrafish model with a four-base pair insertion in the *ahdc1* gene was generated using CRISPR-Cas9 editing (Carvalho et al. 2022). The authors obtained heterozygous *ahdc1*^+/−^ animals, which were fertile and viable, with no obvious phenotypic differences compared to the wild-type ones. The authors proposed that the lower identity of complete AHDC1 protein between human and zebrafish (34%) compared to the identity between human and mouse (94%) could result in a higher tolerance to *ahdc1* mutations in zebrafish (Carvalho et al. 2022). However, they conclude, and we strongly agree, that much more detailed analyses are needed to rule out any possible impact of the mutation in the model. Finally, cellular systems have also been generated. Induced pluripotent stem cell (iPSC) lines were derived by reprogramming peripheral blood mononuclear cells of XIGIS (Carvalho et al. 2022; Yin et al. 2023). These cell lines were characterized to assess their pluripotency and ability to differentiate into the three germ layers and sequenced to confirm the presence of the expected variants (Carvalho et al. 2022; Yin et al. 2023). According to the Authors, these cell models were deposited on the hpscreg.eu platform and may provide a useful tool for studying the pathogenesis of Xia-Gibbs syndrome caused by the *AHDC1* gene. In addition to iPSCs, knock-out *AHDC1* hESCs have been produced in the study of Collier and colleagues (Collier et al. 2022).

## Conclusions

Although the exploration of *AHDC1* has significantly advanced over the past decade, much remains to be uncovered about its functional roles, regulatory mechanisms, and implications in human health and disease. *AHDC1* represents a gene of significant interest and importance, not only in the context of neurodevelopmental disorders like Xia-Gibbs syndrome but also in broader biological and pathological processes such as development, differentiation and schizophrenia. On these grounds, studying *AHDC1* is crucial for several compelling reasons. Its expression patterns highlight its critical role in brain development and function, aligning with the neurodevelopmental abnormalities observed in XIGIS patients. Furthermore, its evolutionary conservation across vertebrates and the preservation of its structure with an intronless coding exon in a specific syntenic organization suggest a fundamental role in vertebrate development. Also, the regulatory mechanisms to which *AHDC1* is putatively subjected, involving both its 5’ and 3’ untranslated regions, hint at sophisticated layers of transcriptional and post-transcriptional control that are yet to be fully understood.

Functionally, AHDC1 is emerging as a crucial player in various biological processes. Its involvement in DNA binding, interaction with other nuclear proteins, and regulation of gene expression positions it as a key regulator within the nucleus. The recent studies linking *AHDC1* to early epithelial morphogenesis, cancer pathogenesis, and metabolic regulation expand its significance beyond neural development, indicating a broader impact on human physiology and disease. The mutations in *AHDC1* associated with XIGIS provide insights into its functional domains and the potential pathogenic mechanisms underlying the disorder. The evidence of truncating and missense mutations suggests that dominant-negative effects or gain-of-function mechanisms may contribute to the disease phenotypes. The variability in phenotypic outcomes observed across different model organisms underscores the complexity of the AHDC1 role and the need for further investigation. The putative propensity of AHDC1 to undergo LLPS introduces an intriguing aspect of its function, potentially linking it to forming membrane-less organelles and, by that, regulating intranuclear processes. This aspect warrants additional exploration to elucidate how LLPS might influence the role of AHDC1 in cellular organization and transcriptional regulation.

Importantly, these insights are crucial for developing strategies to rescue AHDC1 function in disease. These are quintessential examples of precision medicine, as the approach will depend on the specific tissue or brain region exhibiting the primary pathological phenotype in each patient, the timing of disease onset, and the type of mutations involved. Roadblocks to XIGIS therapies are common in most neurodevelopmental disorders:

- Since treatments need to occur postnatally, it is essential to determine how long symptoms remain reversible.
- The minimum brain region requiring intervention must be mapped to guide the targeted delivery of therapeutic molecules (local versus global).
- A detailed analysis of the causative mutation should be conducted, as different therapeutic approaches are required depending on whether the mutation results in dominant-negative effects, haploinsufficiency, or copy number variations (CNVs) where *AHDC1* is hemideleted along with adjacent genes.
- In cases of gain-of-function mutations, allele-specific siRNAs or miRNAs could be customized to patient-specific sequences to inhibit the expression of the mutant allele.
- In situations where the wild-type protein is under-expressed, antisense, long non-coding SINEUP RNAs could be employed to enhance the translation of the residual mRNA and restore the physiological amount and activity of AHDC1 (Espinoza et al. 2020).

Continued investigation into *AHDC1* will undoubtedly unlock further understanding, enhancing our comprehension of fundamental biological processes and paving the way for potential therapeutic interventions, ultimately improving outcomes for individuals affected by AHDC1-related conditions.

## Methods

This review synthesizes data from a range of publicly available resources, experimental studies, and computational tools to comprehensively understand AHDC1. The following methodologies were employed for data collection, analysis, and integration:

1. **Literature search**: PubMed (https://pubmed.ncbi.nlm.nih.gov/) was extensively used for this manuscript, mainly with the advanced literature search focusing on the terms “AHDC1” and “Xia-Gibbs syndrome”.
2. **Data integration**: genomic and transcriptomic data were collected from the following platforms: NCBI GenBank (https://www.ncbi.nlm.nih.gov/genbank/), Ensembl (https://useast.ensembl.org/index.html) and the UCSC genome browsers (https://genome.ucsc.edu/). Expression patterns were analyzed using datasets from the GTEx project (GTEx Consortium 2013) and the BrainSpan Atlas of the Developing Human Brain (Jumper et al. 2021). Evolutionary insights were derived from the Ensembl Compara platform (Herrero et al. 2016).
3. **Protein analysis**: structural insights were obtained from the AlphaFold Protein Structure Database (https://alphafold.ebi.ac.uk/). Functional domains were characterized using tools like InterPro (https://www.ebi.ac.uk/interpro/) (Paysan-Lafosse et al. 2023) and protein alignment databases. Predictions for intrinsically disordered regions (IDRs) and liquid-liquid phase separation (LLPS) propensities were made using Espritz (http://old.protein.bio.unipd.it/espritz/) (Walsh et al. 2012), MobiDB-lite (https://mobidb.org/) (Necci et al. 2017), AIUPred (https://aiupred.elte.hu/) (Erdős and Dosztányi 2024), and catGRANULE2 (Monti et al. 2025).
4. **Comparative Genomics**: the evolutionary history of *AHDC1* was traced by examining its presence and conservation across jawed vertebrates using phylogenetic data from Ensembl (https://www.ensembl.org/). Syntenic relationships with neighbouring genes were analyzed for insights into regulatory conservation.
5. **Clinical data compilation**: case reports and studies detailing *AHDC1* mutations were reviewed. The impact of missense, nonsense, and frameshift mutations was assessed based on their locations and functional implications, utilizing databases such as OMIM (https://www.omim.org/) and SFARI (https://gene.sfari.org/).
6. **Model organism studies**: insights from experimental studies using murine, zebrafish, and cellular models were integrated to highlight functional roles and phenotypic outcomes of *AHDC1* mutations.
7. **Visualization and figure preparation**: figures illustrating genomic organization, expression patterns, and protein domains were created using tools such as the UCSC Genome Browser, BioRender, R and ggplot2.
  By integrating computational analyses, experimental evidence, and clinical data, this review provides a detailed exploration of AHDC1, shedding light on its significance in development, disease mechanisms, and potential therapeutic applications.

## Data Availability

No datasets were generated or analysed during the current study.
